# Supplementary material for: How Large is the Universe of RNA-Like Motifs? A Clustering Analysis of RNA Graph Motifs Using Topological Descriptors
Source: ArXiv. 2025 Jan 8:arXiv:2501.04258v1. Preprint. [Version 1] (PMC11760235)
Supplement: Supplement 1 [file NIHPP2501.04258v1-supplement-1.pdf]

solved RNA database increases in volume. In particular, our striking result that all top RNA-like motifs are decomposable into subgraphs, whereas our top non-RNA-like topologies are irreducible, underscores the modularity and hierarchical nature of biological RNAs. Our work also directly suggests how to design these new RNA-like topologies by combining sequences that correspond to their subgraphs in a build-up strategy as done previously [7, 23].

4.2 Future Directions for RNA Motif Design

Our work indicates that the RNA-like universe is at least 46% and that biological RNA topologies are more likely to contain subgraphs that distinguish them from non-RNA-like structures. Echoing this finding from the perspective of Persistent Spectral Graph (PSG) analysis is that RNA-like graphs tend to exhibit more changes in Betti 0 numbers or the number of connected components, and show an absence of Betti 1 bars or 1-dimensional loops. These findings suggest that the topological features captured by PSG, such as Betti numbers, can be critical indicators of RNA-like characteristics.

Based on these insights, it would be promising to build a comprehensive library of RNA-like graphs characterized by their topological and spectral properties, such as their Betti number profiles and subgraph compositions. This library could serve as a valuable resource for RNA motif design by providing a structured database of potential RNA structures that are more likely to exhibit stable and functional conformations. Researchers could use this library to efficiently screen for novel RNA motifs with desired structural and functional properties, potentially guiding the discovery of new RNA-based therapeutics, biosensors, and regulatory elements. Given the rising popularity and success of AI prediction for systems (such as proteins) where databases are extensive, maintenance of such databases for RNA remains crucial.

In conclusion, our methods and findings provide valuable tools and topological insights for guiding future RNA motif design by highlighting the importance of subgraph patterns and topological features in distinguishing RNA-like and non-RNA-like structures. Our work points to successful build-up approaches for combining nucleotide sequences of corresponding subgraphs of the target graph, as done using RAG [23]. Our library of RNA-like graphs, informed by PSG analysis and enhanced by machine learning, helps the discovery and development of novel RNA motifs, paving the way for innovative RNA-based technologies.

Code and Data availability

The code and data for the feature and clustering algorithms are available at the public repository PSGRNA-Clustering. The RNA inverse folding using dual graph representations package is available at Dual-RAG-IF.

Supporting Information

The Supporting Information is available for:

- S1 Supplementary Methods
  - S1.1 Basic topological concepts
    - S1.1.1 Topological concepts
    - S1.1.2 Combinatorial Laplacians
  - S1.2 Persistent spectral graphs
  - S1.2 Clustering algorithms

S1.3.1 k-means clustering

S1.3.2 Mini-batch k-means clustering

S1.3.3 Gaussian mixture model (GMM)

S1.3.4 Hierarchical clustering using Ward's method

S1.3.5 Spectral clustering

S1.3.6 Balanced iterative reducing and clustering using hierarchies (Birch)

S1.4 Evaluation Metrics

S1.4.1 Silhouette score and homogeneity score

S1.4.2 Sensitivity of binary clusters

S1.5 Designed samples

Acknowledgment

Support from the National Institutes of Health, National Institute of General Medical Sciences Award R35-GM122562, National Science Foundation Awards (DMS-215177 and DMS-2330628) from the Division of Mathematical Sciences, and Philip-Morris USA Inc to T.S. is gratefully acknowledged. R.W. is grateful for the support from the Simons Foundation and the Simons Center for Computational Physical Chemistry (SCCPC) at New York University. R.W. also thanks Dr. Shuting Yan for helpful discussions.
